# Prevalence of Dyspnea among Patients Attending the Emergency Department of a Tertiary Care Hospital: A Descriptive Cross-sectional Study

**DOI:** 10.31729/jnma.4582

**Published:** 2019-10-31

**Authors:** Anmol Purna Shrestha, Roshana Shrestha, Sanu Krishna Shrestha, Alok Pradhan, Samjhana Basnet

**Affiliations:** 1 Department of General Practice and Emergency Medicine, Dhulikhel Hospital, Dhulikhel, Kavre, Nepal

**Keywords:** *chronic obstructive pulmonary disease*, *dyspnea*, *emergency medicine*, *shortness of breath*

## Abstract

**Introduction:**

Dyspnea is a common presenting complaint in the emergency department worldwide and a diagnostic challenge for emergency physicians. Our study aims to find the prevalence of dyspnea among patients attending emergency department in our hospital.

**Methods:**

A descriptive cross-sectional study was conducted in the emergency department of Dhulikhel hospital from May 2019 to July 2019 after ethical approval from the institutional review committee. Total 1200 samples were collected by consecutive sampling method. All patients were triaged in the emergency department as a part of regular protocol. The participants were included in the study after obtaining an informed consent from the patient or caretaker (if the patient were not able to provide it). Point estimate at 95% confidence interval was calculated along with frequency and proportion for binary data. The statistical analysis was done using R version 3.5.3 (2019-03-11).

**Results:**

The prevalence of dyspnea among patients attending emergency department of a tertiary care hospital was 107 (8.9%) (4.6%-13.2%) at 95% confidence interval. The patients triaged into red, orange and yellow categories were 14 (13.1%), 50 (46.7%) and 43 (40.2%) respectively. Median age was 64 years and 74 (69%) were ≥60 years. Sixty-seven (62.6%) were females and 40 (37.4%) were males. Forty-four (41.1%) arrived by ambulance. Most commonly associated symptoms were cough and fever 59 (51.1%) and 44 (41.1%) respectively.

**Conclusions:**

The prevalence of dyspnea among patients attending emergency department of our hospital is higher compared to that of other studies. This warrants structured and prompt management of dyspnea for quality improvement.

## INTRODUCTION

Dyspnea or Shortness of Breath (SOB) is a common presenting complain reported in the Emergency Department (ED) worldwide.^1-3^ It can be a diagnostic challenge in emergency setting because it may be the primary manifestation of cardiovascular, respiratory, metabolic, neuromuscular disorders or obesity.^4-6^ Though many studies are available from high income countries regarding dyspnea in EDs, little is known about the prevalence, distribution, diagnostic approach, treatment modalities and outcome of dyspnea in EDs in our population. Further research is needed to improve and standardize the quality of care in ED without inadvertent wastage of limited resources in a low-income country like Nepal. The information obtained from this study may be utilized to provide further direction to cost-effective modalities for quality enhancement of patient care in ED.

The primary objective of this study was to find out the prevalence of dyspnea among patients attending emergency department of tertiary care hospital of Nepal.

## METHODS

A descriptive cross-sectional study was conducted in the ED of Dhulikhel Hospital after approval from the institutional review committee from May 2019 to July 2019. All patients were triaged in the emergency department as a part of regular ED protocol. The triage nurse informed the concerned investigator if a patient more than 18 years of age with subjective or objective symptoms of dyspnea presented to ED. The participants were included in the study after obtaining an informed consent from the patient or caretaker (if the patient were not able to provide it). Dyspnea secondary to trauma, poisoning/drug overdose and participants not willing to give consent were excluded.

The following formula was used to calculate the sample size.

$$\text{n}=\frac{{\text{Z}}^{\text{2}}\times \text{p}\times \left(\text{1}-\text{p}\right)}{{\text{e}}^{\text{2}}}$$

$$\begin{array}{l} ={\text{(1.96)}}^{\text{2}}\times 0.5\times 0.5/{\text{(0.05)}}^{\text{2}}\\ =384.1\\ =385 \end{array}$$

where,

n = minimum sample size
Z= 1.96 for Confidence Interval at 95%
p= 50%
e= margin of error as 5%

Taking 4% as a non-response rate, the sample size becomes 400. Consecutive sampling was done so calculated sample size was tripled. Total 1200 samples were collected. Data was collected using the pre-designed proforma and entered in Microsoft Excel. The statistical analysis was done using R version 3.5.3 (2019-03-11). Point estimate at 95% Confidence Interval was calculated along with frequency and proportion for binary data.

## RESULTS

**Figure 1:**
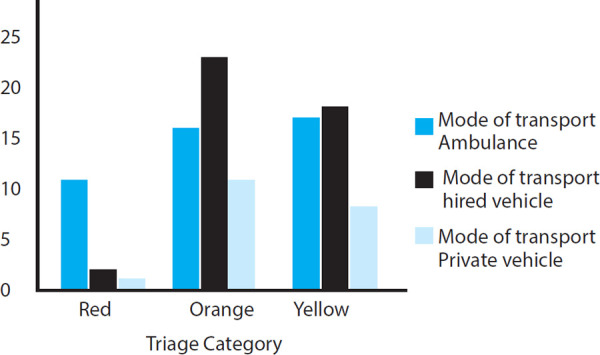
Mode of transport according to the triage category.

The prevalence of dyspnea among 1200 patients who presented to the emergency department of our hospital was 107 (8.9%) (4.6%-13.2%) at 95% Cl. Median age of the patients presenting with primary complaint of dyspnea was 64 years (IQR=54-72) with 74 (69%) patients ≥60 years. Sixty-seven (62.6%) of them were females. Forty-four (41%) patients arrived by ambulance. The patients triaged into red, orange and yellow categories were 14 (13.1%), 50 (46.7%) and 43 (40.2%) respectively. Eleven (79%) patients triaged as red category followed by 15 (30%) and 4 (39%) patients triaged as orange and yellow category arrived via ambulance (Figure 1).

Twenty-one (19.6%) patients were current smokers. Sixty-eight (63.3%) patients had a history of COPD with 17 (15.9%) patients under domiciliary oxygen. Fifty-five (51.4%) patients had a history of visits to the emergency department within the previous one year. Five (4.7%) patients presented within previous one week, 11 (10.3%) within 1-4 weeks, 25 (23.4%) within 1-6 months, 7 (6.5%) 6-12 months and 7 (6.5%) more than one year. Thirty-four (61.8%) patients had previous hospital admission (Table 1).

**Table 1:** Patient characteristics.

Variable	n (%)
Age in years	
Median (IQR)	64 (54-73)
Gender	
Female	67 (62.6)
Male	40 (37.4)
Education	
No formal education	60 (56)
Primary level	23 (21.5)
High school	12(11.2)
Higher education	12(11.2)
Triage category	
Red	14(13.1)
Orange	50 (46.7)
Yellow	43 (40.2)
Green	0 (0)
Mode of arrival	
Ambulance	44 (41)
Hired vehicle	43 (40.2)
Private vehicle	20 (18.7)
Smoking status	
Non-smoker	41 (38.3)
Active smoker	21 (19.6)
Past smoker	45 (42.1)
Comorbidities	
Chronic Obstructive	68 (63.6)
Pulmonary Disease	9 (8.4)
Diabetes	8 (7.5)
Cardiac	7 (6.5)
Hypertension	4 (3.7)
Asthma	3 (2.8)
Alcoholism	9 (8.4)
Others	21 (19.6)
None	
Duration of dyspnea in days	
Median (IQR)	3 (2-6)
Previous visit to ED	55 (51.4)
Use of domiciliary oxygen	17 (15.9)

Associated symptoms were cough, fever, chest pain and swelling. Complete blood count and renal function test were the most frequent investigations performed in 96 (89.7%) and 93 (86.9%) patients respectively. Eighty five (79.4%) and 82 (76.6%) patients had their ECG and CXR done respectively. Lung ultrasound was done in 11 (10.3%) cases. Symptoms were attributed to diagnosis involving respiratory 56 (52.3%), respiratory and cardiovascular 14 (13.1%), metabolic 12 (11.2%), respiratory with others 10 (9.3%), neuropsychiatric 7 (6.7%), cardiovascular 4 (3.7%) and gastrointestinal 4 (3.7%) systems as illustrated in (Table 2).

**Table 2:** Diagnosis of the cases.

Variable	n (%)
ED Diagnosis	
Respiratory	56 (52.3)
Respiratory and cardiovascular	14 (13.1)
Metabolic	12 (11.2)
Respiratory with others	10 (9.3)
Neuropsychiatric	7 (6.7)
Cardiovascular	4 (3.7)
Gastrointestinal	4 (3.7)

Forty-five (45%) were admitted. Forty-one patients (38.3%) were admitted in the medical ward and 3 patients (2.8%) were admitted in ICU/HDU. The length of stay of the patients at the ED before the patients were decided for disposition from ED was between one and twenty hours. Eight (57.1%) patients from the red category were admitted while 30 (69.8%) patients from the yellow category were discharged.

## DISCUSSION

Our study found that dyspnea is a common presentation to ED with prevalence of 8.9%. Forty two percent of these patients required admission in hospital, making them a high consumer of acute healthcare resources. The most common diagnoses were involving respiratory system (52.3%). Importantly, in 22.4% of cases an undifferentiated mixed cause [respiratory and cardiovascular (13.1%) or respiratory with others (9.3%) was found. This has implications for service planning, protocol-based care and training.

We followed a syndromic approach targeting patients with dyspnea rather than aiming at a single disease. This approach is important as patients who present to the ED do not come with a predefined diagnosis. This poses a great deal of challenge to the ED clinicians who need to be equipped with ample knowledge and protocol-based approach to determine the likely cause and its severity for further appropriate treatment.

A large study was done by Kelly et al. in Southeast Asia and Australasian cohort study investigating diagnoses and outcome of ED patients with dyspnea.^2^ In the context of Nepal, we found no study done in ED setting to describe the burden of patients presenting to ED with shortness of breath as a primary complaint. However, we have a few studies done on certain disorders like COPD and asthma.^7^ COPD is the third among the diseases causing years lived with disability (YLDs), the second most common cause of death after IHD in Nepal and fourth common cause of premature deaths.^8^ In a study done considering the exposure to biomass smoke, those exposed had higher prevalence of respiratory symptoms, urban dwellers (who were exposed to higher ambient air pollution) were more at risk of having productive cough.^7^

In the context of Nepal, this is the first study of this kind that reports the prevalence, caseload, causes and the outcome of patients with dyspnea at ED. The prevalence of dyspnea in our study was 8.9% which is comparable to the study done in Europe (7.4%),^9^ India (6.3%)^10^ and the Asia-Pacific region (5.2%).^2^ In a study done in India,^10^ it was found that in a busy ED, dyspnea ranked third in the list of the most common presentations (next to fever and renal colic), highlighting the fact that we need to have robust data in our low resource settings in order to mobilize and utilize the limited resources according to the caseloads.

In another large study conducted in EDs by Kelly et al.^11^ they observed that patients transported by ambulance with shortness of breath have high comorbidity and high hospital admission rate. Similar result was demonstrated by our study with 79% of the patients triaged into red category arrived via ambulance. It was notable finding that most patients (58.9%) did not utilize ambulance services.

Our study found that most common diagnoses involved respiratory and mixed systems (respiratory, cardiovascular and other symptoms) which is similar to the findings of the study by Kelly et al. where >60% were accounted for by patients with heart failure, lower respiratory tract infection or COPD, but there was a wide range of diagnosis. In our study, we found COPD to be the most common comorbidity with 15% of these patients already under domiciliary oxygen use. Dyspnea and chronic cough are well established common presentations of COPD. Acute exacerbation of COPD is a leading cause of morbidity and mortality in South Asian countries.^12^

Significant proportion of dyspneic patients had critical vital signs during presentation such as tachycardia (53.3% with pulse ≥120), hypoxia (75.5% with oxygen saturation <90%) and abnormal blood pressure (19.6%). This was reflected by the fact that 83.2% patients required oxygen therapy, 6.5% required non-invasive ventilation, 2.8% required invasive ventilation and 14% patients required critical care services. Likewise, the high utilization of ABG is not surprising given the high proportion of sick patients. Chest X-ray and ECG were performed in the majority of patients. POCUS is a valuable yet underutilized tool in ED setting which is demonstrated by only 10.3% patients undergoing bedside ultrasound. The reason behind this may be that the lung ultrasound is an emerging technique and limited number of clinicians are trained in its application. Lung ultrasound has shown to have higher accuracy in routine clinical evaluation of patients with dyspnea for differentiating causes of dyspnea in the emergency department.^13-15^ The authors have concluded that the strategy can be adopted even in resource limited settings.

We realize that further research is the need of time in this area to improve and standardize the quality of care in ED without wasting the limited resources in a low-income country like Nepal. The data from this study has implications for further ED staff education. Our study may serve as an important foundation to base future research activity given the scarcity of data in ED setting. Some innovative future researches could be regarding approach and management to mixed etiologies of dyspnea, quality improvement in the management of COPD and further training and full utilization of the emerging diagnostic modalities like lung ultrasound, which is feasible and cost-effective even in resource limited setting like ours.

Our study does not represent the EDs of whole Nepal, however the population benefited from the ED services represent both urban and rural population. The diagnosis categories were based on the treating ED physician’s opinion, which may have differed with further evaluation and investigation. The study did not follow the outcome of the patients admitted to inpatient facilities.

## CONCLUSIONS

The prevalence of dyspnea among patients attending emergency department of our hospital is higher compared to that of other studies. The study showed that most patients with dyspnea had involvement of either respiratory or respiratory with cardiovascular or other system and further development of the protocols for assessment, investigation and treatment can be based upon the findings of this study.

## Conflict of Interest:

None.
